# Poisoning by Purity: What Stops Stereocomplex Crystallization in Polylactide Racemate?

**DOI:** 10.1021/acs.macromol.2c02067

**Published:** 2023-01-21

**Authors:** Jiaming Cui, Shu-Gui Yang, Qilu Zhang, Feng Liu, Goran Ungar

**Affiliations:** † Shaanxi International Research Center for Soft Matter, State Key Laboratory for Mechanical Behavior of Materials, Xi’an Jiaotong University, Xi’an710049, China; ‡ Department of Materials Science and Engineering, Sheffield University, SheffieldS1 3JD, U.K.

## Abstract

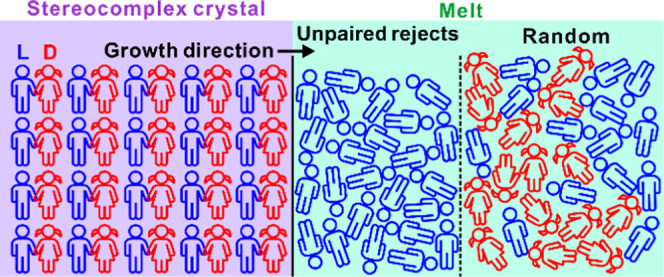

Formation of stereocomplex crystals (SC) is an effective way to improve the heat resistance and mechanical performance of poly(lactic acid) products. However, at all but the slowest cooling rates, SC crystallization of a high-molecular-weight poly(l-lactic acid)/poly(d-lactic acid) (PLLA/PDLA) racemate stops at a high temperature or does not even start, leaving the remaining melt to crystallize into homochiral crystals (HC) or an SC–HC mixture on continuous cooling. To understand this intriguing phenomenon, we revisit the SC crystallization of both high- and low-molecular-weight PLLA/PDLA racemates. Based on differential scanning calorimetry (DSC), supplemented by optical microscopy and X-ray scattering, we concluded that what stops the growth of SC is the accumulation of the nearly pure enantiomer, either PDLA or PLLA, that is rejected from the SC ahead of its growth front. The excess enantiomer is a result of random compositional fluctuation present in the melt even if the average composition is 1:1. The situation is more favorable if the initial polymer is not fully molten or is brought up to just above the melting point where SC seeds remain, as proven by DSC and X-ray scattering. Moreover, we find that not only is SC growth poisoned by the locally pure enantiomer but also that at lower temperatures, the HC growth can be poisoned by the blend. This explains why SC growth, arrested at high temperatures, can resume at lower temperatures, along with the growth of HC. Furthermore, while some previous works attributed the incomplete SC crystallization to a problem of primary nucleation, we find that adding a specific SC-promoting nucleating agent does not help alleviate the problem of cessation of SC crystallization. This reinforces the conclusion that the main problem is in growth rather than in nucleation.

## 1 Introduction

Stereocomplex crystals (SC) comprising a racemic or near-racemic mixture of enantiomeric poly(l-lactic acid) (PLLA) and poly(d-lactic acid) (PDLA) helices endow the racemic blend outstanding thermal and mechanical properties.^1−9^ The melting temperature of SC reaches ∼230 °C, approximately 50 K higher than that of homochiral crystals (HC)^10^ of the pure enantiomers. Formation of SC has shown great potential for improving the heat resistance of poly(lactic acid) (PLA) products in practical applications, e.g., avoiding the collapse of PLA beverage cups filled with hot drinks. Considering the current emphasis on environmentally friendly plastics, boosting thermal properties and thus widening the usage of the biodegradable PLA are of particular interest.^11−13^ Efforts to boost the formation of SC in PLA have been ongoing. On the basis of elaborate exploration, many strategies have been reported to promote the formation of SC. These include the synthesis of stereo-block PLLA-*b*-PDLA,^14−16^ melt blending at a temperature between the melting points of SC and HC,^17,18^ adding nucleating agents,^19,20^ flow-induced crystallization,^6,21,22^ high temperature annealing,^2,5^ etc. In spite of all this effort, crystallization of pure SC still remains an unsolved challenge, especially in a high-molecular-weight (HMW) PLLA/PDLA racemate.^23−26^

Previous studies have shown that HC crystallization prevails over SC crystallization for HMW racemates with weight-average molecular weight *M*_w_ ≥ 4 × 10^4^ g/mol under regular crystallization conditions.^27,28^ A number of suggestions have been made to explain the suppression of SC in the HMW PLLA/PDLA racemate. In the 1990s, Tsuji et al. proposed that microscopic phase separation may occur in precursor solution during solvent evaporation in film casting of the HMW PLLA/PDLA racemate; such separation would form unfavorable conditions for SC crystallization while favoring HC growth.^27^ The effect of phase separation on SC crystallization has been further addressed by Li et al.^29^ and Lan et al.^30^ More recently, Hu et al. applied Tammann analysis on crystallization of the PLLA/PDLA racemate using flash differential scanning calorimetry (DSC).^31^ They reported that increasing the molecular weight of the PLLA/PDLA racemate suppresses the nucleation rate of SC but has little impact on HC nucleation. These observations were attributed to monomer units of higher-molecular-weight chains, having an increased probability of encountering their neighbors belonging to the same chain, which suppressed intermolecular nucleation required for SC crystallization.

In spite of the considerable amount of past experimental work, it is clear that significant controversy still remains as to the cause of suppressed SC crystallization in the HMW PLLA/PDLA racemate. Careful inspection of some of the previously reported cooling DSC traces reveals an unusual phenomenon: after taking off at high temperatures (∼180 °C), SC crystallization stops, with crystallization taking off again on further cooling, but this time in the HC form.^32−34^ Such behavior is difficult to explain by reduced primary nucleation. We have been puzzled as to why SC crystallization stops at high temperatures, way above the glass transition, where chain diffusion should be easy, active crystal growth faces are present in abundance, and free-energy difference between the melt and crystal (the “driving force”) continuously increases as temperature decreases. The only precedents to such inversion of crystallization rate gradient with temperature known to us are the cases of self-poisoning, first observed in ultralong *n*-alkanes above the extended-to-folded-chain^35,36^ and once-to-twice-folded-chain growth transitions.^37^ Poisoning of crystal growth faces by impurities is a well-known and well-studied occurrence in crystallization in general.^38^ For example, some drugs are made amorphous to increase their solubility in the body, and polymer impurities can be added to deliberately poison their surface and prevent crystallization.^39^ Effect of impurities on poisoning growth of polymer crystals is also not a new subject.^40,41^ Self-poisoning is at the heart of crystallization of any flexible polymer but reveals itself only in special circumstances in the form of growth rate minima. Stopping crystal growth of a complex caused by poisoning by one of its pure components, reported in this work, is a new phenomenon. It is interesting scientifically and deserves to be studied theoretically and/or by simulation. The effect is also of considerable industrial interest as it seems to be the major obstacle in the formation of the highly desirable temperature-resistant PLA stereocomplex under industrially relevant cooling rates.

In this work, PLLA/PDLA racemic blends, one HMW and one low-molecular-weight (LMW), were used to study SC crystallization in continuous cooling using DSC, X-ray scattering, and polarized optical microscopy. We propose an explanation of the interesting phenomenon of SC crystallization ceasing at high temperatures and then resuming as HC or SC + HC crystallization on continuous cooling. This explanation introduces a new concept in polymer crystallization, which we refer to as “poisoning by purity”.

## 2 Experimental Section

### 2.1 Materials

HMW and LMW PLLA and PDLA were purchased from Jinan Daigang Biomaterial Co., Ltd. They were used without further purification. According to gel permeation chromatography (see Figure S1 and Table S1 in SI), *M*_w_ values of HMW PLLA and PDLA were 8.2 × 10^4^ g/mol, and the polydispersity (PDI) of the HMW pair was 1.7. The *M*_w_ values of LMW PLLA and PDLA were 2.0 × 10^4^ and 2.1 × 10^4^ g/mol, respectively, and the PDI was 1.3 and 1.6, respectively. 1,4-Dioxane was purchased from Sinopharm Chemical Reagents Co., Ltd. and used as received. The nucleating agent β-NA arylamide derivative (TMB-5) with a chemical structure similar to *N*,*N*′-dicyclohexyl-2,6-naphthalenedicarboxamide was kindly supplied by the Fine Chemicals Department of the Shanxi Provincial Institute of Chemical Industry.

### 2.2 Preparation of the PLLA/PDLA Racemate

PLLA/PDLA 1:1 blends were prepared as follows: PLLA and PDLA were separately dissolved in 1,4-dioxane (mp 12 °C) at 50 °C with a concentration of 0.1 g/mL. Ten milliliters of both solutions was then mixed together under stirring for 20 min at 50 °C. To ensure that no concentration gradient develops during solvent evaporation, the mixed solution was frozen by quenching in liquid nitrogen. The frozen PLLA/PDLA racemate was then freeze-dried under vacuum at 0 °C for 12 h to sublime off the solvent.

### 2.3 Characterization

#### 2.3.1 DSC

**Figure 1:**
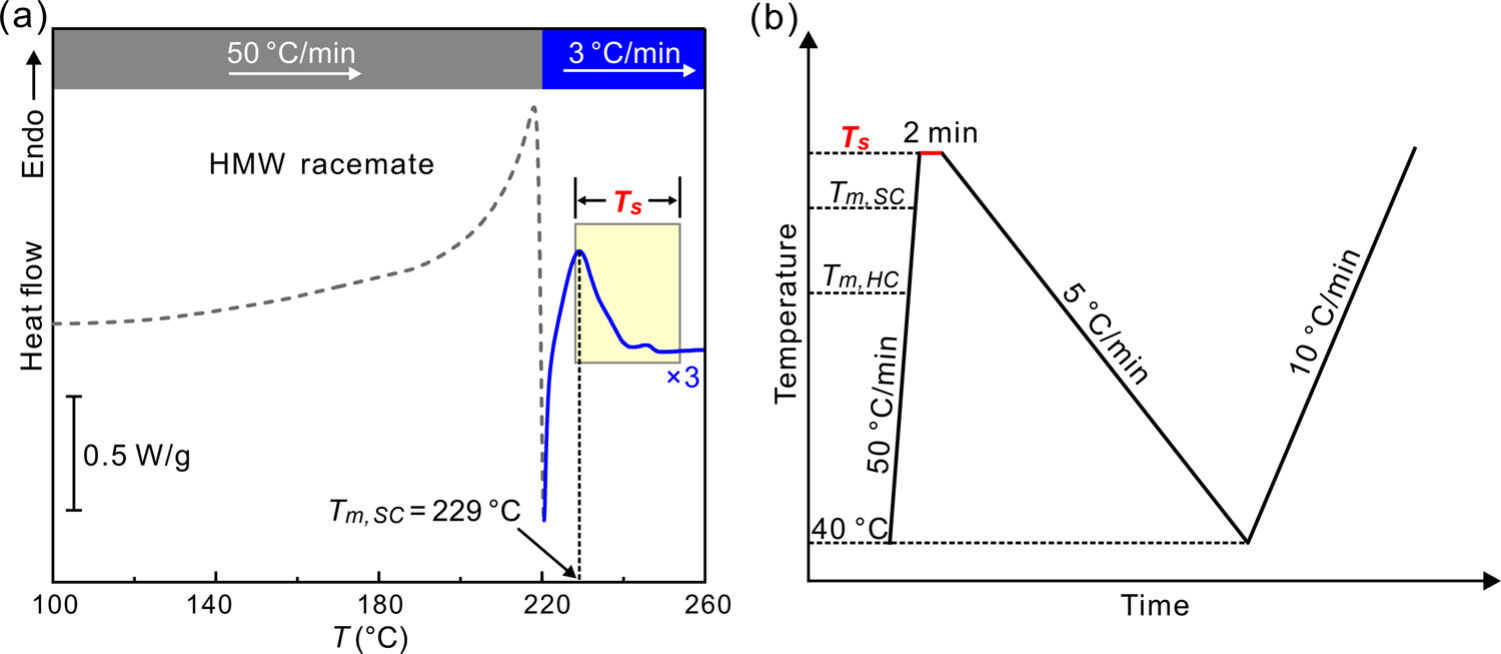
(a) DSC heating curve of the HMW PLLA/PDLA racemate containing exclusively SC. Up to 220 °C, the heating rate was 50 K/min (gray dashed curve), and for 220–260 °C, it was 3 K/min (blue curve). The scan is performed to determine the range of seeding temperatures (highlighted in yellow). (b) Thermal profile of the experiments.

The key experiments in this study are DSC crystallization runs of racemic PLLA/PDLA during cooling. The “self-seeding” method is used throughout.^42^ Cooling runs were performed from a range of “seeding” temperatures (*T*_s_), as defined by the yellow-shaded area in Figure 1a. The preliminary DSC scan in Figure 1a was performed to estimate how much, if any, sample is still crystalline at *T*_s_. The HMW racemate containing exclusively SC was first heated to 220 °C at a rate of 50 K/min to minimize degradation and then continuously heated to 260 °C at 3 K/min. For subsequent DSC crystallization experiments on the HMW racemate, the *T*_s_ values were chosen within the yellow-shaded range above the peak melting point. The chosen *T*_s_ range for the LMW racemate is shown in Figure S2 of the Supporting Information.

Crystallization and subsequent melting behavior of the PLLA/PDLA racemate were investigated as defined by the temperature program depicted in Figure 1b. A TA DSC250 instrument was used and calibrated with indium. A fresh sample was used in each run to avoid excessive thermal degradation. First, the blend was heated to *T*_s_ at a rate of 50 K/min and annealed for 2 min, and then the cooling scan to 40 °C at 5 K/min was recorded, followed by a reheating scan to 260 °C at 10 K/min. %Crystallinity of SC was calculated, as usual, as , where Δ*H*_m_ and Δ*H*_m_^0^ are the respective melting enthalpies of the SC form in the sample and in fully crystalline stereocomplex equal to 142 J/g.^43^

#### 2.3.2 Wide-Angle X-ray Scattering (WAXS)

To evaluate how much SC remained at different *T*_s_, WAXS of the HMW PLLA/PDLA racemate was recorded, using Cu Kα radiation, on an Anton Paar SAXSpoint 2.0 instrument equipped with an Eiger 2-panel detector and a temperature-controlled capillary holder. To prepare the sample for WAXS measurement, the HMW racemate was melted in capillary at 250 °C and then quickly transferred to a vacuum oven with a preset temperature of 160 °C and held there for 20 min to crystallize in SC form. For the temperature-variable WAXS measurement, the sample was heated at a rate of 3 K/min to melt. The exposure time of each pattern was 1 min. %Crystallinity of SC (*X*_c_) was obtained using , where the shape of *A*_amorp_ was determined from a rapidly quenched fully amorphous PLA sample. An example of the curve resolution of a WAXS profile is shown in Figure S3 of the Supporting Information.

#### 2.3.3 Polarized Optical Microscopy (POM)

Crystalline morphology of the HMW PLLA/PDLA racemate was observed using an Olympus BX51 microscope equipped with a Linkam LTS420E hot stage and a T95-HS controller. The sample was heated to various *T*_s_ between two glass slides and annealed for 2 min before recording images during cooling the sample to room temperature at a rate of 5 K/min.

## 3 Results

### 3.1 DSC Cooling Experiments on LMW and HMW Racemates

**Figure 2:**
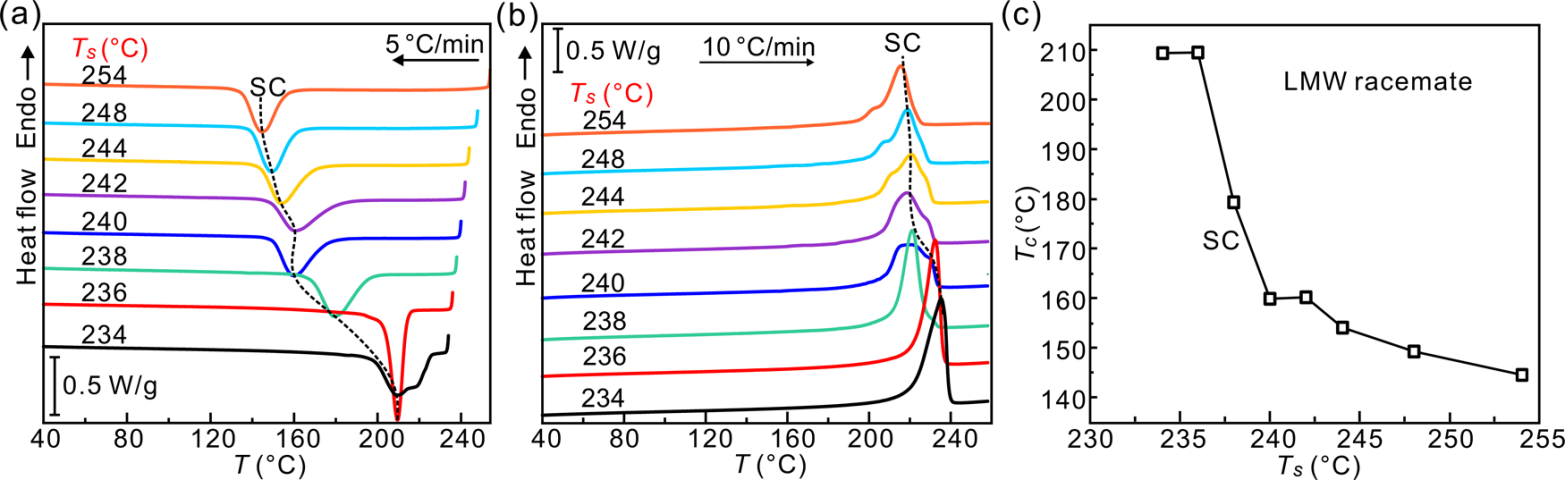
(a) DSC curves for the LMW racemic mixture on cooling at 5 K/min from different *T*_s_. (b) Second heating runs at 10 K/min. (c) Peak temperature of SC crystallization exotherms of 5 K/min cooling thermograms as a function of *T*_s_.

Figure 2a shows the DSC cooling curves of the LMW PLLA/PDLA racemate at 5 K/min. A single exotherm is observed, regardless of *T*_s_. Meanwhile, all of the subsequent DSC heating curves show a single endotherm above 200 °C (Figure 2b), corresponding undoubtedly to melting of SC. For reference, the melting point of HC is to be found at temperatures below 175 °C. Thus, we can confirm that for all seeding temperatures, cooling of the LMW racemate at 5 K/min invariably results in SC. The peak crystallization temperature (*T*_c_) of SC is plotted as a function of *T*_s_ in Figure 2c. *T*_c_ is found to decrease with increasing *T*_s_. The sharp drop in *T_c_* between *T*_s_ = 236 °C and *T*_s_ = 240 °C probably indicates that below 236 °C, the “seeds” are actually the remaining crystallites.

**Figure 3:**
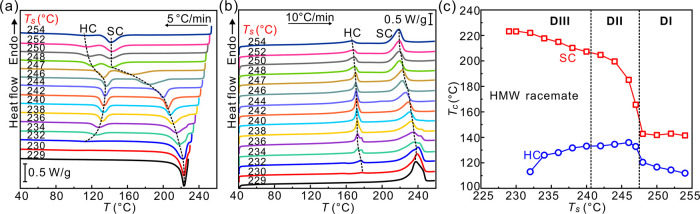
DSC (a) cooling (5 K/min) and (b) subsequent heating (10 K/min) thermograms of the HMW racemate following a 2 min dwell at different *T*_s_. (c) Variation of crystallization temperature of SC and HC vs *T*_s_ obtained from panel (a).

For the HMW PLLA/PDLA racemate, two exotherms appear in the DSC cooling curves when *T*_s_ > 230 °C (Figure 3a). One is in the range from 225 to 140 °C, depending on *T*_s_. The other is around 130 °C. Meanwhile, two melting peaks at ∼170 and ∼230 °C can be seen in subsequent heating thermograms (Figure 3b), corresponding to melting of HC and SC, respectively. The crystallization enthalpies (Δ*H*_c_) of the exotherms on cooling are consistent with the enthalpies (Δ*H*_m_) of melting of SC and HC from the respective endotherms on heating in Figures S4 and S5 of the Supporting Information. This correspondence implies that the exotherms above 140 °C come from crystallization of SC, while those around 130 °C are from crystallization of HC. Figure 3c shows the dependence of SC and HC *T*_c_ on *T*_s_. With regard to *T*_s_, the entire temperature region can be divided into three domains. In the high *T*_s_ range I (DI) where *T*_s_ ≥ 248 °C, *T*_c_ of SC (∼140 °C) remains invariant with *T*_s_ because 248 °C is high enough to completely erase crystal memory. Interestingly, the 140 °C SC crystallization exotherm is accompanied by an HC crystallization exotherm at a somewhat lower temperature, which gradually broadens and fades as *T*_s_ is increased further above 250 °C. In the mid-temperature range II of 242 °C ≤ *T*_s_ ≤ 247 °C (DII), *T*_c_ of SC is seen to increase steeply with decreasing *T*_s_. As in the LMW racemate, the increase in *T*_c_ of SC is attributed to the presence of seeds. Significantly however and unexpectedly, unlike in the LMW racemate, the SC exotherm is relatively small, while the second sharp exotherm is present around a constant temperature of 130 °C, attributed to the crystallization of HC. The hiatus in SC crystallization observed in this (DII) *T*_s_ range is a key observation in this work to be tested and discussed further below. In the low-*T*_s_ range III (DIII, *T*_s_ ≤ 240 °C), the SC crystallization exotherm is dominant, and its temperature increases slowly with decreasing *T*_s_, presumably due to the presence of an increasing fraction of still unmolten crystallites at *T*_s_. At the same time, the HC exotherm around 130 °C fades away.

**Figure 4:**
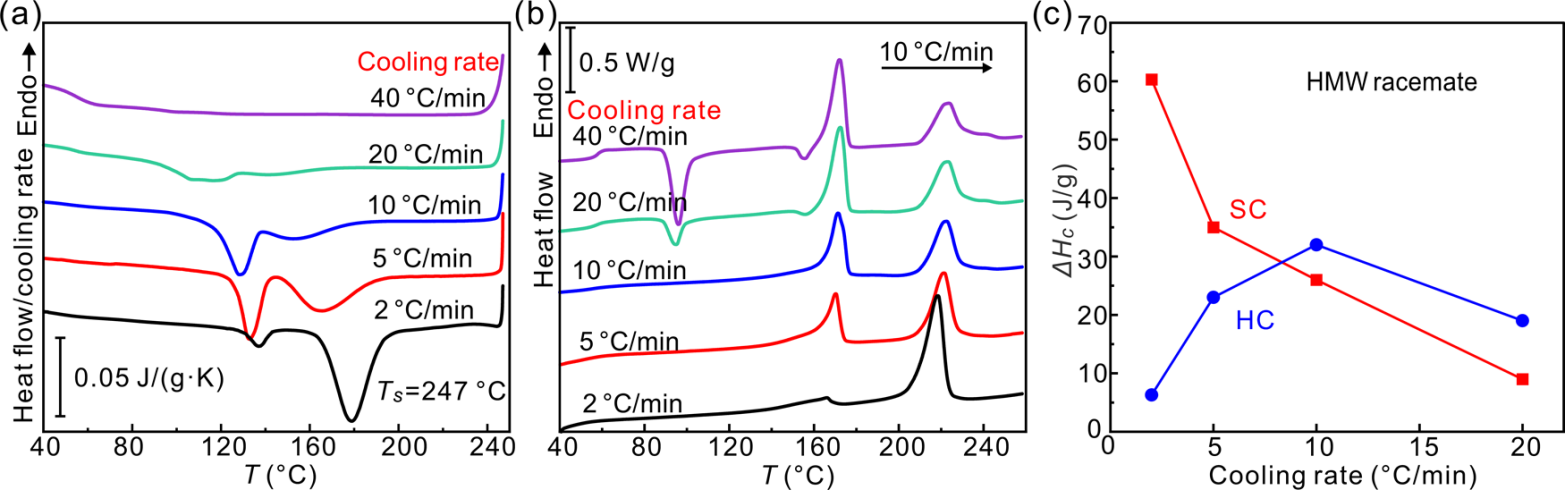
(a) Normalized DSC cooling curves of the racemic HMW blend recorded at different rates after annealing at 247 °C for 2 min. (b) Subsequent heating curves with a rate of 10 K/min. (c) Plot of Δ*H*_c_ of SC and HC on cooling as a function of the cooling rate. For curve resolution applied, see Figure S6 of the Supporting Information.

To understand the above crystallization behavior, DSC cooling runs of the HMW PLLA/PDLA racemate were done at different rates after holding for 2 min at the DII-range temperature *T*_s_ = 247 °C. As shown in Figure 4a,c, the HC exotherm initially increases at the expense of the SC exotherm as the cooling rate increases. This is verified by the areas of the melting peaks on subsequent heating (Figure 4b). The nearly 5-fold increase in crystallinity of SC with a cooling rate reduction from 40 to 2 K/min indicates that the hiatus in SC crystallization described above at 5 K/min is not due to complete cessation but only to a significant slowdown in crystallization.

### 3.2 Crystallization of the HMW Racemate with Added TMB-5 Nucleating Agent

**Figure 5:**
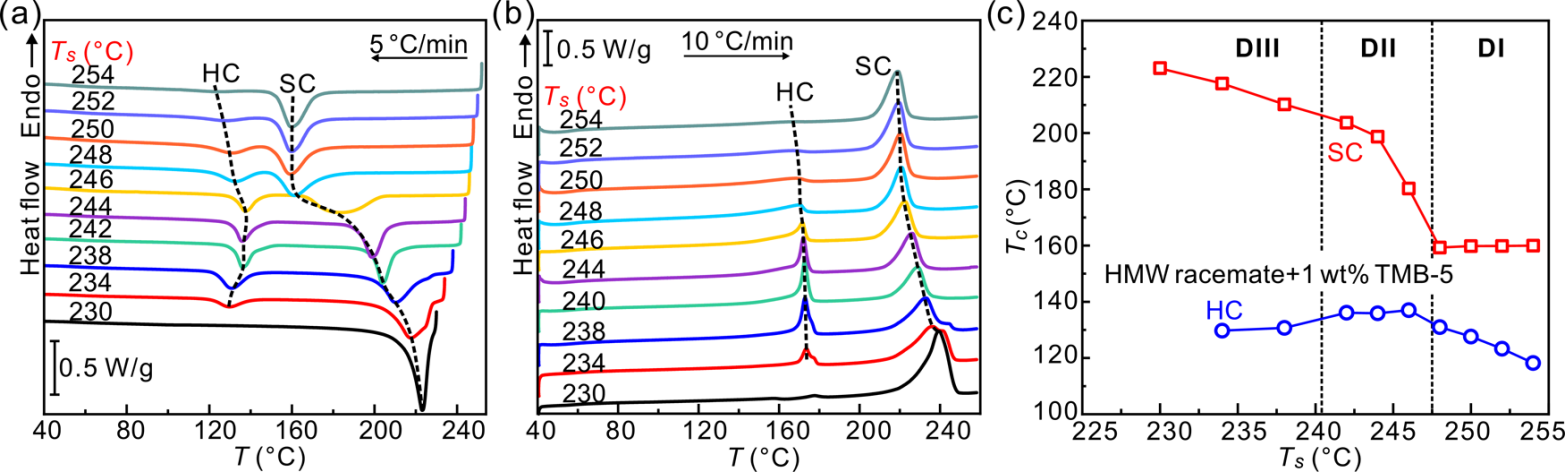
DSC (a) cooling and (b) heating thermograms of the HMW PLLA/PDLA racemate containing 1 wt % TMB-5. The cooling rate is 5 K/min, and the heating rate is 10 K/min. (c) Plot of SC (red square) and HC (blue circle) crystallization temperature of the HMW racemate with 1 wt % TMB-5 as a function of *T*_s_.

A recent study has suggested that SC nucleation of the HMW PLLA/PDLA racemate is suppressed because, due to the prevalence of intramolecular nucleation, there is an above-average local concentration of homochiral monomer units belonging to the same polymer molecule.^31^ To test whether this could be the reason for the premature cessation of SC crystallization observed in our study, we added to our HMW racemate the nucleating agent TMB-5, designed to specifically promote SC nucleation.^19^ The related DSC cooling and subsequent heating runs of such racemate are shown in Figure 5a,b. Similar to the situation in the HMW racemate without the nucleating agent, two crystallization peaks are seen in the cooling run, one in the 225–150 °C range and the other around 130 °C. These can again be assigned to SC and HC crystallization, respectively, after comparing the Δ*H*_c_ and Δ*H*_m_ of SC and HC (see Figures S7 and S8 of Supporting Information). One can still see that SC crystallization ceases and is subsequently followed by HC crystallization at a lower temperature. In Figure 5c, *T*_c_ of SC and HC is plotted as a function of *T*_s_. In comparison with the neat HMW PLLA/PDLA racemate (see Figure S9 of the Supporting Information), *T*_c_ of SC in TMB-5-containing racemate is increased by 20 °C in the DI range (*T*_s_ ≥ 248 °C). The increase in *T*_c_ of SC demonstrates that TMB-5 is an effective nucleating agent for SC. And yet the extra SC nuclei created by TMB-5 could not prevent the premature cessation of SC crystallization in the cooling cycles. We thus conclude that the problem is not in crystal nucleation but in their growth. One can also see that the SC crystallization temperature remains almost unchanged in the lower *T*_s_ ranges DII and DIII, irrespective of the addition of TMB-5. Furthermore, the *T*_c_ of HC is again affected by the formation of SC in a similar way as in the neat racemate.

### 3.3 Temperature Evolution of WAXS of the HMW Racemate on Heating

**Figure 6:**
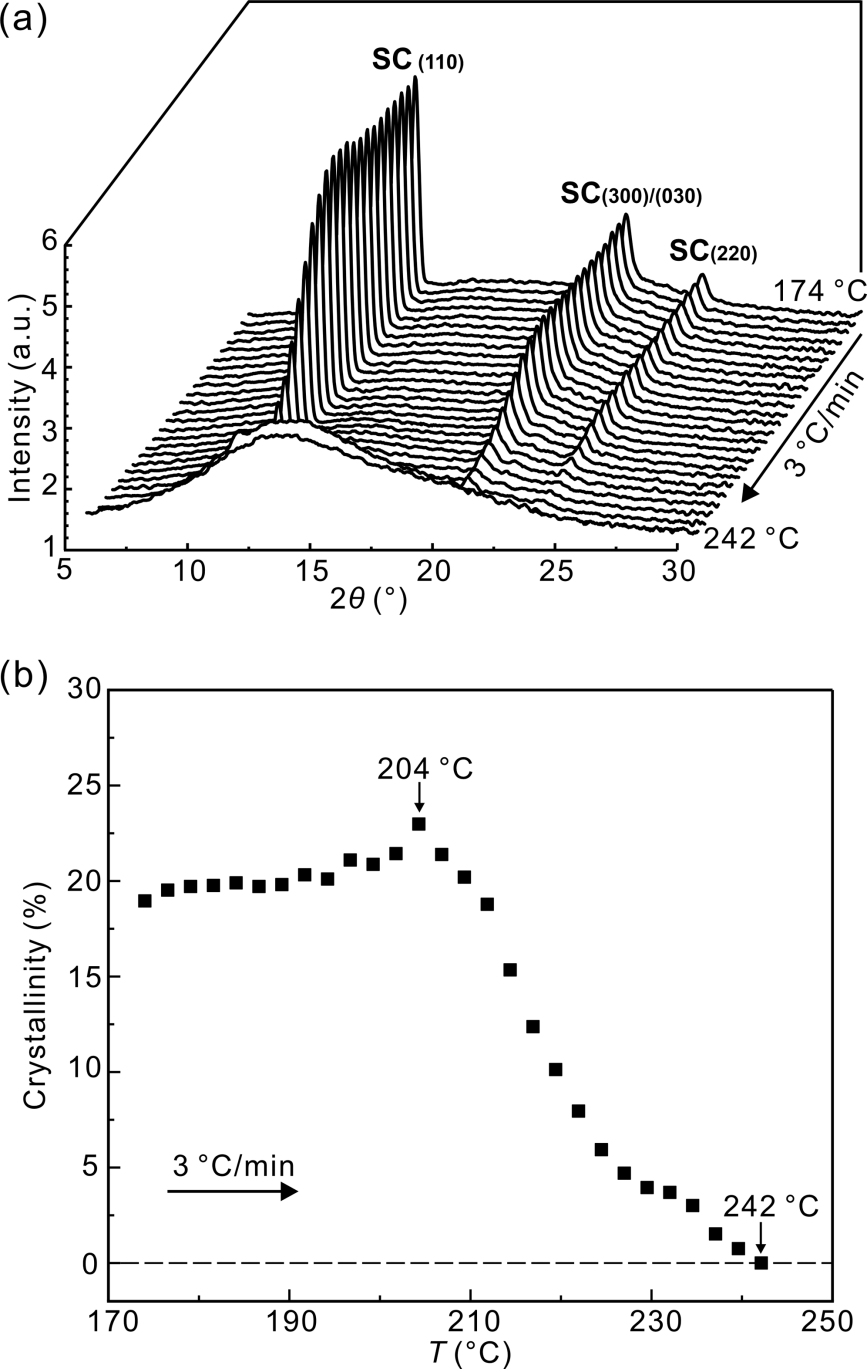
(a) WAXS profiles of the HMW PLLA/PDLA racemate collected during 3 K/min heating to check for possible existence of crystallinity at *T*_s_. Prior to the experiment, the sample was annealed at 160 °C to complete the crystallization of SC. (b) Change of SC crystallinity in heating.

To evaluate how much SC crystallinity survives in the HMW racemate at different *T*_s_, WAXS was recorded during a 3 K/min heating run. Figure 6a shows the collected WAXS profiles. One can see that the characteristic reflections of SC, i.e., SC_(110)_, SC_(300)/(030)_, and SC_(220)_, gradually fade away with increasing temperature and disappear completely at 242 °C. The WAXS evidence of some still unmolten SC at *T*_s_ ≤ 240 °C agrees with the borderline between DIII and DII being at around 241 °C as determined by DSC (Figure 3d). X-ray crystallinity *X*(*T*) is plotted in Figure 6b (see Figure S3 for an example of curve resolution). The initial increase to 23% at 204 °C is attributed to the effect of annealing. The fact that detectable *X*(*T*) → 0 at 242 °C while the SC exotherm still continues to broaden and move to lower temperatures with increasing *T*_s_ between 242 and 247 °C (Figure 3a) may be taken as evidence of some kind of seeds persisting beyond crystal melting.

### 3.4 Crystal Morphology of the HMW Racemate on Cooling from Different *T*_s_

**Figure 7:**
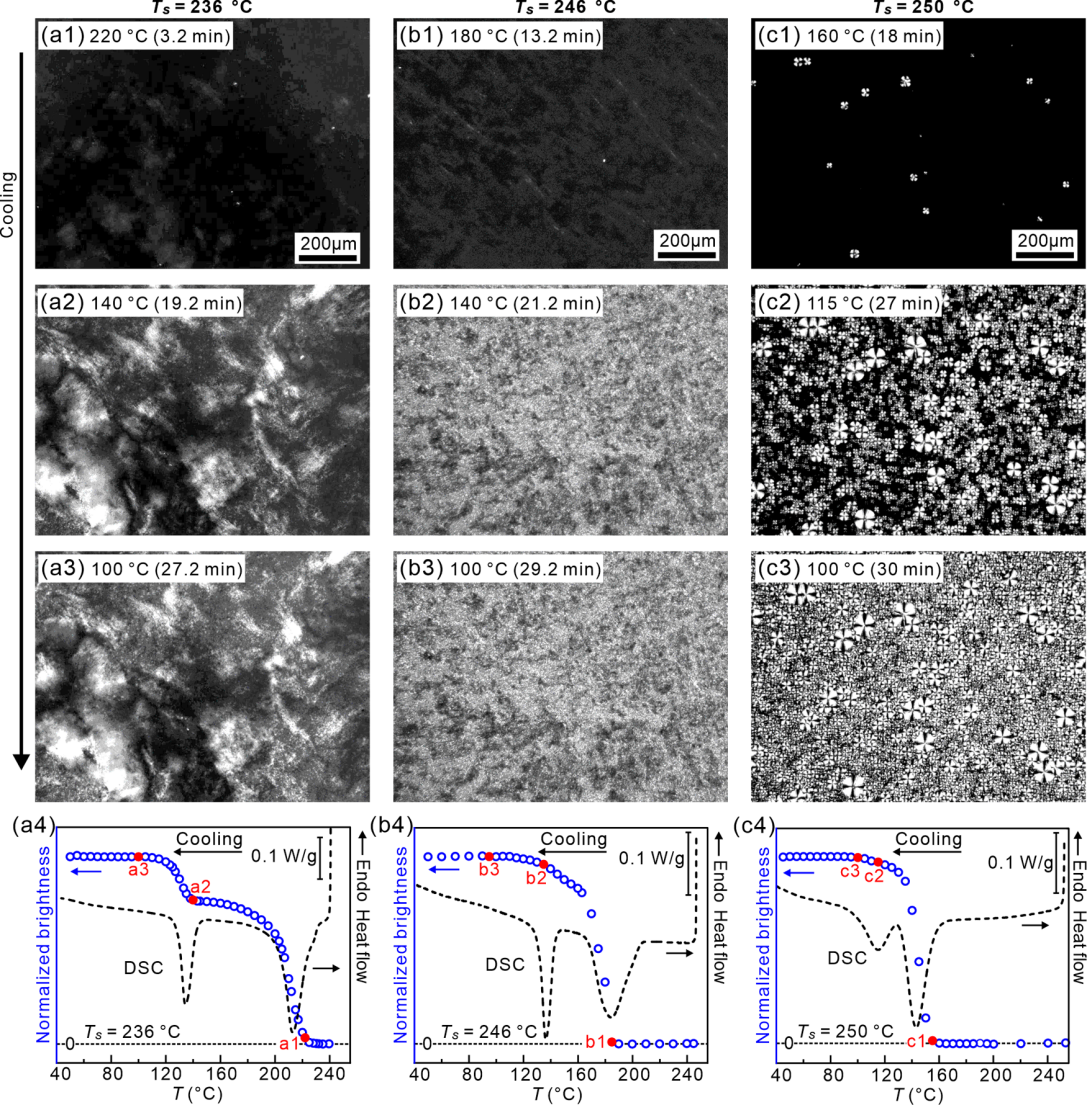
POM micrographs of the HMW PLLA/PDLA racemate recording during cooling (5 K/min) from different *T*_s_. (a1–a3) *T*_s_ = 236 °C; (b1–b3) *T*_s_ = 246 °C; (c1–c3) *T*_s_ = 250 °C. (a4–c4) Change in integrated brightness of POM images vs temperature during the 5 K/min cooling runs for *T*_s_ of (a4) 236, (b4) 246, and (c4) 250 °C.

Next, crystalline morphologies of the HMW racemate annealed at three different *T*_s_, 236, 246, and 250 °C, were studied by POM. We note that no birefringence was observed at the start of cooling for any of the three selected seeding temperatures (see Figure S10 of the Supporting Information). On cooling from *T*_s_ = 236–200 °C, the fog-like birefringent texture appeared over the entire field of view (Figure 7a1). The “fog” corresponds to submicron SC crystalline domains nucleated on crystalline fragments surviving after annealing at 236 °C. Similar birefringent pattern has been described by Fillon et al.^44^ and Lorenzo et al.^45^ On cooling from *T*_s_, the birefringent texture brightens up in two distinct steps (Figure 7a1–a3): first upon cooling from 220 to 140 °C, attributed to crystallization of SC, and second on cooling from 140 to 115 °C, corresponding to crystallization of HC. These two steps match exactly the two DSC exotherms, as shown in Figure 7a4.

Increasing *T*_s_ to 246 °C, a much greater density of small spherulites appear from the nonbirefringent melt at 180 °C (Figure 7b1). As noted in the above WAXS (Figure 6), after annealing at *T*_s_ = 246 °C, there are no SC crystalline Bragg reflections, but the racemate may still contain seeds. When the temperature is reduced further, the spherulites become brighter (Figure 7b2,b3). The increase in brightness is related to SC and HC crystallization, in agreement with the DSC cooling curve (Figure 7b4).

For *T*_s_ = 250 °C, rare spherulites are nucleated at ∼160 °C (Figure 7c1). Upon further cooling, they grow slowly; meanwhile, smaller spherulites appear in the surrounding melt (Figure 7c2,c3). According to DSC (Figure 7c4), these correspond to both SC (first) and HC (second). As already observed by DSC, for this highest *T*_s_ of 250 °C, SC spherulites first appear at a much lower temperature than those for *T*_s_ = 246 °C (Figure 7b).

## 4 Discussion

The most intriguing question raised by the experimental data in this work is why, in cooling runs started at temperatures *T*_s_ between 232 and 247 °C, crystallization of the SC seizes prematurely, only to resume at a lower temperature in either SC or HC form or in both. What stops SC crystallization only halfway to its final crystallinity? As already mentioned, the temperature is high above *T*_g_ and the racemate is well mixed by freeze-drying. With decreasing temperature, the polymer crystal growth rate should increase exponentially, unless the increasing viscosity on approaching *T*_g_ (i.e., a rapidly decreasing pre-exponential factor β) dominates the kinetics. This is certainly not the case here where, according to classical Lauritzen–Hoffman nucleation theory,^46^ the dominant temperature-dependent factors are either exp(−4*b*σσ_e_/Δ*F* × *kT*) or exp(−2*b*σσ_e_/Δ*F* × *kT*), depending on whether the growth surface is smooth (regime I) or rough (regime II). The respective full expressions for the growth rate (*G*) are^47^
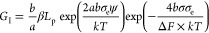
1
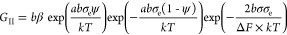
2With all other parameters being constants except for the relatively slowly varying β and *T*, a steep increase in *G* with increasing supercooling Δ*T* should be ensured, as usual, by the driving force Δ*F* increasing nearly linearly with Δ*T* and thus steeply increasing the attachment survival factor exp(−*K*/Δ*F* × *kT*). As mentioned in the Introduction section, the only examples where, far from *T*_g_, such increase is reversed are the cases of self-poisoning (SP).^35−37,48^ In most SP examples so far, the retardation in *G* with increasing Δ*T* occurs above the transition from thick lamella growth to thinner lamella growth, where such transitions are quantized due to certain preferred values of lamellar thickness. In long monodisperse *n*-alkanes, these transitions are from extended- to once-folded^35,36^ or from once- to twice-folded-chain growth.^37^ In sharp low-molecular-weight PEO fractions, it is again from extended- to once-folded chains.^49,50^ In segmented polydisperse polyethylenes with regularly spaced substituents such as Br or acetal groups, the transitions are between lamellar thicknesses corresponding to an integer number of such chain segments.^51,52^ In all of these cases just above the growth transition temperature, *G* of the thicker lamella is obstructed by depositions of shorter, nearly stable chain stems with finite but still reasonably long lifetime. These are cases where Lauritzen–Hoffman theory fails, but fine grain theories like that of Sadler^53^ or related approaches by the rate equation^35,36^ or simulations^48,53,54^ reproduce the kinetics reasonably well, at least qualitatively.

However, in the polydisperse racemate of PLLA and PDLA, there is no reason to assume preferred discrete values of lamellar thickness. One could perhaps also consider another type of self-poisoning, where a less stable polymorph but with a lower free-energy barrier for crystallization might compete with the growth of the more stable crystal form; such a situation may account for certain cases of “polymorphic” SP.^55^ We have considered the possibility that unstable depositions of HC could poison the growth of the stable SC above the HC melting point. However, we rejected that possibility because the “minimum” in growth rate, while expected and observed to be sharp in genuine SP cases, is very broad here, with a no-growth gap up to 70 K wide in the present case (see Figure 3a). Furthermore, the “classic” SP could not explain why the “minimum” and the retardation effect itself disappear in the LMW polymer racemate.

**Figure 8:**
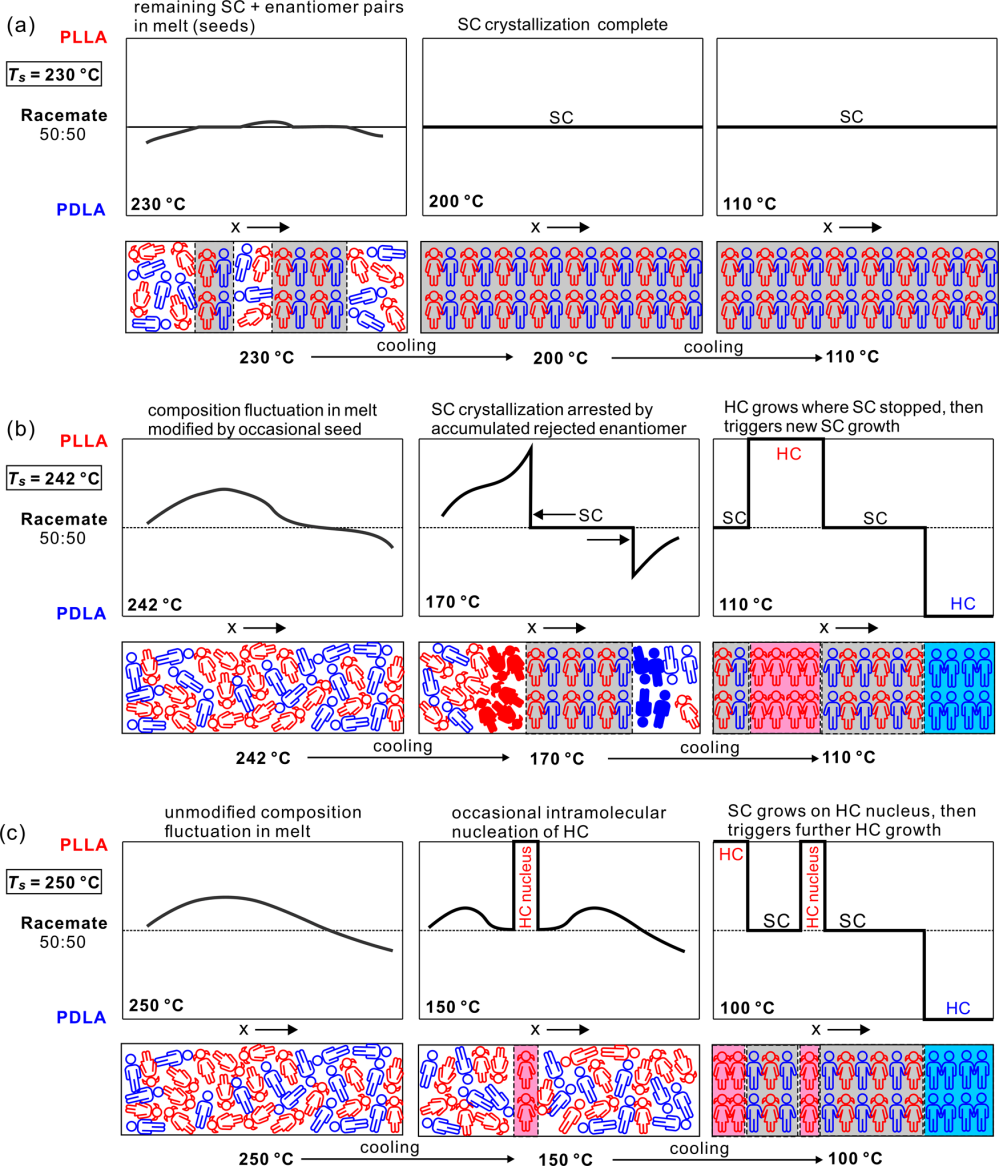
Schematic diagram showing the events during cooling of the HMW PLLA/PDLA racemate after brief annealing at three different *T*_s_. The top row of each subfigure shows spatial variation of an enantiomer ratio at three temperatures based on DSC cooling thermograms in Figure 3a. The bottom rows depict the structure with the “boys” and “girls”, symbolizing molecular segments of PDLA and PLLA, respectively. The filled figures in the sketch for *T*_s_ = 242 °C, *T* = 170 °C symbolize the enantiomeric excess rejected ahead of the growing SC, blocking its further growth. Compare with DSC cooling traces in Figure 3a.

We propose instead another explanation for the present finding, which we may call “poisoning by purity”. Although the stereocomplex of PLA is known to be quite tolerant to deviations from the ideal 1:1 enantiomeric ratio,^56,57^ the deviations may exceed that tolerance. There are certainly natural local fluctuations in the enantiomeric ratio (ER) in the melt, in which the growth of SC may amplify. We explain our idea using a pictorial scheme in Figure 8.

In the first row (Figure 8a), depicting the situation in the low-*T*_s_ range DIII, we consider the cooling experiment starting from *T*_s_ = 230 °C. Because not all crystallites had melted at that temperature, crystallization of SC proceeds uninhibited as the growth front finds ER within tolerable bounds everywhere it spreads. However, at the higher *T*_s_ of 242 °C (intermediate temperature range DII, Figure 8b), there are insufficient seeds to dampen the ER fluctuations everywhere in the melt as efficiently as at the lower *T*_s_. SC growth starts in areas with ER closest to 1:1, such as at the remaining seeds. However, as the growth front encounters domains with an excess of one or the other enantiomer, it rejects it, so that the excess enantiomer accumulates ahead of the front. The resulting high local enantiomeric purity effectively becomes impurity for the SC, poisoning its surface and stopping its growth. Yet the temperature is still too high for those enantiopure domains to form HC. The whole process can be likened to a mating game, where, in spite of the average human population containing an equal number of males and females, there will be local fluctuations resulting in parts of the population remaining single. The mating problem could be resolved if there is sufficient mobility in the system, which explains why, in the more mobile LMW racemate, SC crystallization proceeds to completion for all *T*_s_. That the “mating” process is diffusion-controlled is also obvious from the results in Figure 4. Cooling from *T*_s_ = 247 °C at a slower rate of 2 K/min, most crystallization succeeds to produce SC, while at rates higher than 10 K/min, SC remains in minority.

Crystallization of the *T*_s_ = 242 °C HMW sample on cooling at 5 K/min resumes only when the temperature drops to the *T*_c_ of HC, i.e., ∼140 °C, allowing the accumulated enantiopure regions to crystallize either as PDLA or PLLA homochiral crystals.

The situation seems even more complicated if *T*_s_ is increased higher (temperature range DI). At *T*_s_ = 250 °C (Figure 8c), there are no seeds left and the composition fluctuations in the melt are purely statistical. At a cooling rate of 5 K/min, no calorimetrically noticeable crystallization takes place until reaching ∼140 °C. Interestingly, while increasing the *T*_s_ from 242 to 247 °C progressively depresses and broadens the SC crystallization exotherm (Figure 3a), for *T*_s_ ≥ 248 °C, the exotherm sharpens drastically again and remains at a constant temperature with further increase in *T*_s_. The subsequent DSC heating runs (Figure 3b) confirm that the said exotherm indeed comes from crystallization of SC, while the second lower temperature exotherm appearing at a slightly lower temperature represents HC crystallization. The question is now why had the SC exotherm not continued to broaden and move to still lower temperature with increasing *T*_s_. Continuous broadening and shift to a lower temperature would be expected especially considering that 120 °C is about the position of the crystallization rate maximum for PLA, below which the decreasing chain mobility (diminishing β-factor) retards the crystallization even more.

To explain the unexpected sharpness and constant position of the ∼140 °C SC exotherm in cooling runs with *T*_s_ ≥ 248 °C, we propose that the first nuclei exceeding the critical size are those of HC, not SC. The roles are now reversed. As sketched in Figure 8c, the small HC soon gets trapped by the opposite enantiomer rejected to their growth face. The wrong enantiomer impurity blocking the HC growth means, however, that an ER closer to 1:1 is established in the surrounding, now enabling the growth of SC. This multiple negative feedback, with the ER oscillating between the values favorable to the growth of either SC or HC, allows both crystal types to grow within a relatively narrow temperature range. However, one should note that the overall crystallinity achieved with such high *T*_s_ is significantly lowered (see Figure S5 of the Supporting Information). Incidentally, it may be the case that HC nucleation occurs more readily, even at significantly smaller supercooling than SC nucleation, because of a preference for intramolecular nucleation.^31^

Here, we also need to mention that the situation with the stereocomplexation is actually more complex than sketched in our simple scheme. As shown by the recent X-ray work by Tashiro et al., SC can contain anything between the ER of 7:3 to 3:7, with an up–down pair of homochiral chains able to replace an antichiral pair, where needed.^56,57^

Regarding the POM images in Figure 7, the abundance of nuclei and the short distance the crystallization front can move before encountering the accumulated enantiopure poisoning barrier in cooling runs with *T*_s_ ≤ 247 °C is consistent with the birefringent “fog”, i.e., unresolved submicrometer spherulites, as seen in Figure 7a,b. For *T*_s_ = 250 °C, no “fog” is seen, but a few small isolated and slow-growing spherulites appear already at 160 °C, too small a volume fraction to be registered by DSC. The different but generally small-sized spherulites seen at lower temperatures include both SC and HC, one cleaning the melt for the growth of the other.

## 5 Conclusions

In this work, we have reported a new addition to the already rich list of complex phenomena in polymer crystallization, i.e., poisoning not by impurities but self-poisoning by native polymer chains but of wrong chirality, rejected to the growth front and thus unable to be incorporated to the growing streocomplex.

## Supporting Information Available

GPC data of HMW and LMW PLA polymers; DSC heating run of the LMW racemate; curve resolution of the WAXS profile; resolution of exotherm of the HMW racemate and HMW racemate + 1 wt % TMB-5; comparison of enthalpies of exotherms and endotherms of the HMW racemate and HWM racemate + 1 wt % TMB-5; *T*_c_ of SC and HC in the HMW racemate + 1 wt % TMB-5 vs *T*_s_; POM micrographs of the HMW racemate (PDF)
