# Supplementary material for: Poisoning by Purity: What Stops Stereocomplex Crystallization in Polylactide Racemate?
Source: Macromolecules. 2023 Jan 21;56(3):989–98. doi: 10.1021/acs.macromol.2c02067 (PMC9933539; doi:10.1021/acs.macromol.2c02067)
Supplement: Supplementary file 1 — ma2c02067_si_001.pdf [file ma2c02067_si_001.pdf]

Supporting Information

Poisoning by Purity: What Stops Stereocomplex Crystallization in Polylactide Racemate?

Jiaming Cui[1], Shu-Gui Yang[1,], Qilu Zhang[1], Feng Liu[1], and Goran Ungar[1,2,*]*

[1] Shaanxi International Research Center for Soft Matter, State Key Laboratory for Mechanical Behavior of Materials, Xi'an Jiaotong University, Xi'an 710049, China

[2] Department of Materials Science and Engineering, Sheffield University, Sheffield S1 3JD, UK

1. GPC of HMW and LMW PLLA and PDLA polymers

Molecular weight distribution of LMW and HMW PLLA and PDLA was measured by GPC. Chloroform was used as the mobile phase. The GPC curves are shown in **Figure S1**. More information about molecular weight and PDI (*Đ*) are provided in **Table S1**.

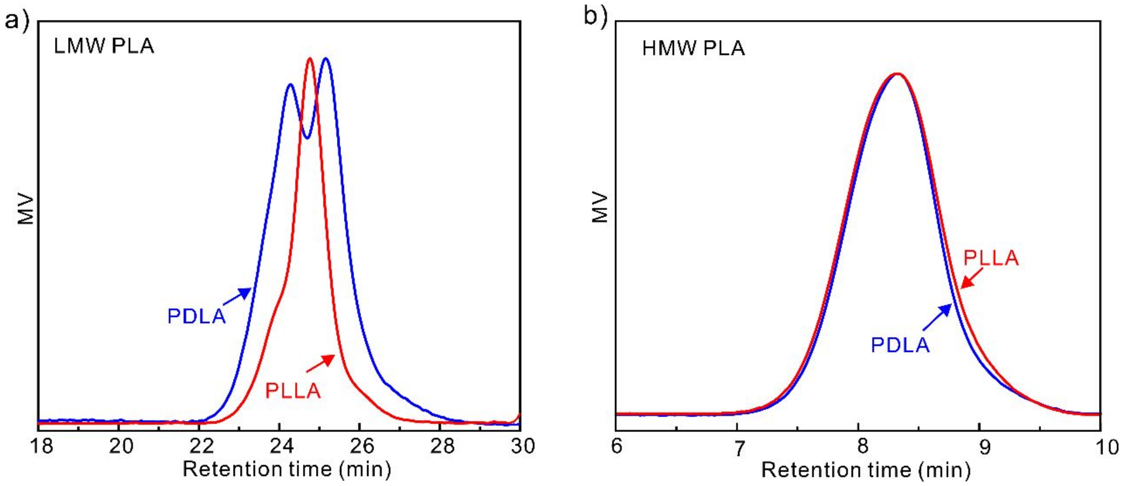

Figure S1. Gel permeation chromatograms of LMW and HMW PLLA and PDLA.

Table S1. Molecular weights of LMW and HMW PLLA and PDLA. Polystyrene was used as reference standards.

		M_w (g/mol)	M_n (g/mol)	*Đ*
LMW	PLLA	20000	16000	1.3
	PDLA	21000	13000	1.6
HMW	PLLA	82000	48000	1.7
	PDLA	82000	49000	1.7

2. T_s range of LMW PLLA/PDLA racemate

DSC heating curve of LMW racemate is shown in **Figure S2**. To avoid degradation, the sample was firstly heated to 220 ˚C at a rate a 50 K/min (grey dashed curve) then continuously heated to 260 ˚C at a rate of 3 K/min (blue curve). The selected T_s range is shaded yellow in the heating thermogram shown in **Figure S2**. The melting peak temperature of SC was calibrated by indium.

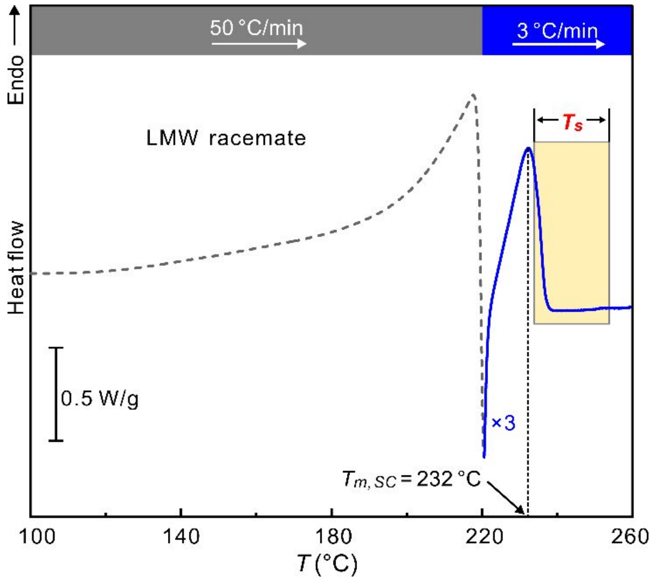

Figure S2. DSC heating curve of LMW PLLA/PDLA racemate. For temperature lower than 200 ˚C, heating rate was 50 K/min (grey dashed curve); for 200 – 260 ˚C, it was 3 K/min (blue curve).

3. Curve resolution of WAXS profiles of HMW PLLA/PDLA racemate

To evaluate the crystallinity of SC in HMW racemate at different temperature, the WAXS profiles were resolved into individual Bragg components and the amorphous scattering curve using Origin. WAXS profile recorded at 220 ˚C is shown as an example in **Figure S3**.

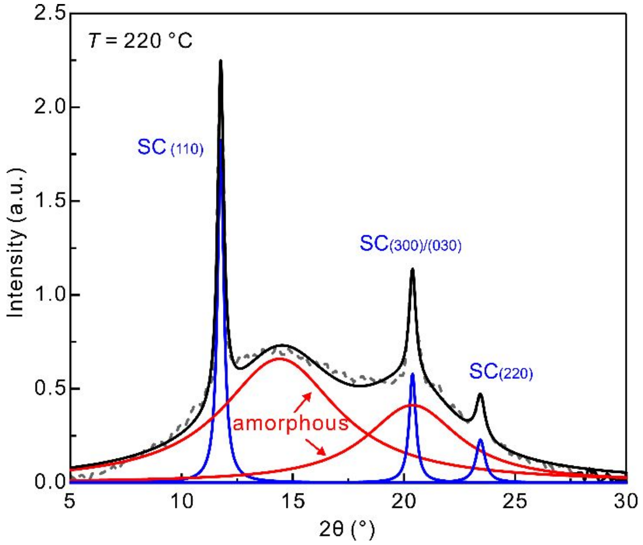

Figure S3. Peak-fitting of WAXS profile collected at 220 ˚C in heating.

4. Assignment of exotherm of SC and HC in HMW racemate with different T_s

For $T_s \leq 247$ ˚C, enthalpies of higher and lower temperature exotherm are consistent with melting enthalpies of SC and HC melting peak, respectively. However, for $T_s \geq 248$ ˚C, the higher and lower temperature exotherms are partially overlapped with each other. It was assumed that the higher and lower temperature exotherm respectively corresponds to SC and HC (**Figure S4**). To confirm this, the exotherm was decomposed by peak-fitting using modified Gaussian function[S1,S2] and the enthalpies were compared with endotherms in second heating, as shown in **Figure S5**, higher and lower temperature exotherm corresponds to SC and HC, respectively.

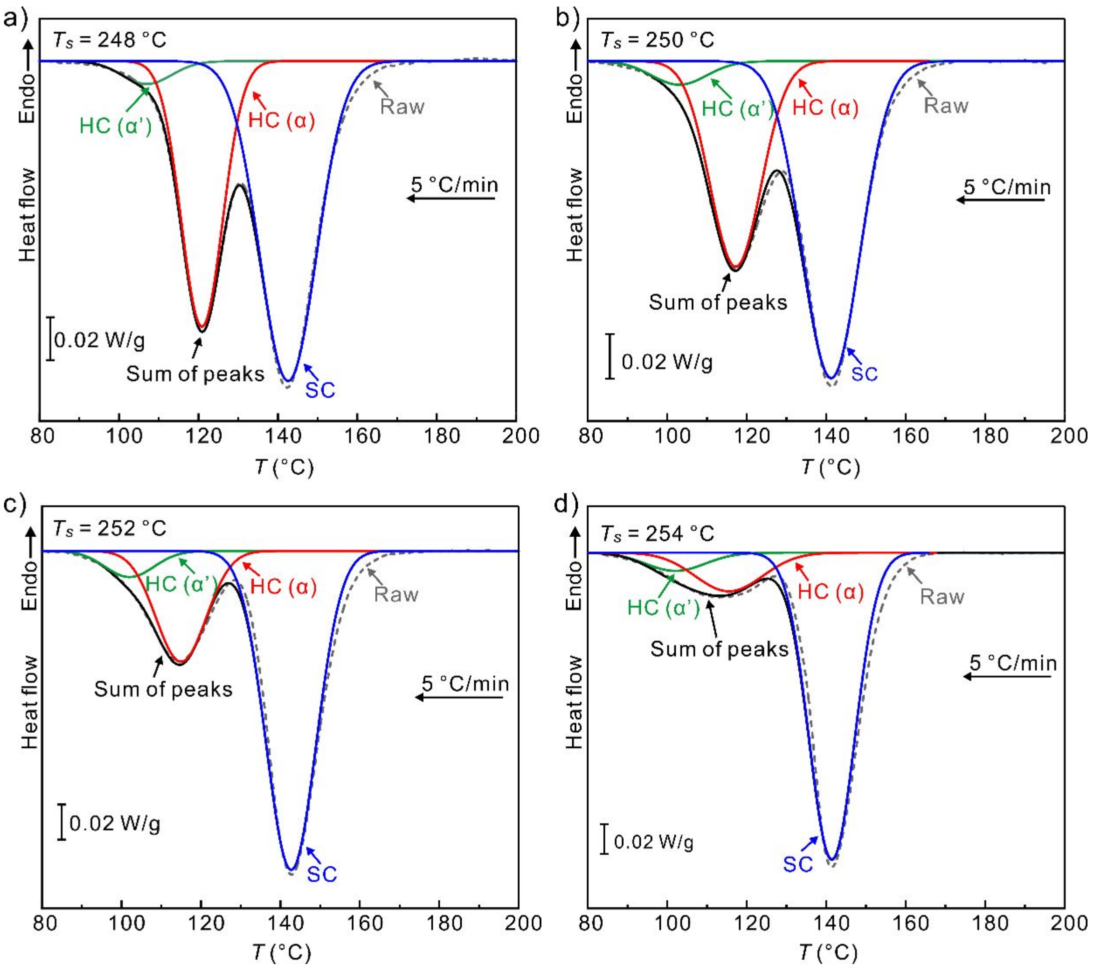

Figure S4. Resolution of exotherms of HMW PLLA/PDLA racemate on cooling from T_s of (a) 248, (b) 250, (c) 252 and (d) 254 °C.

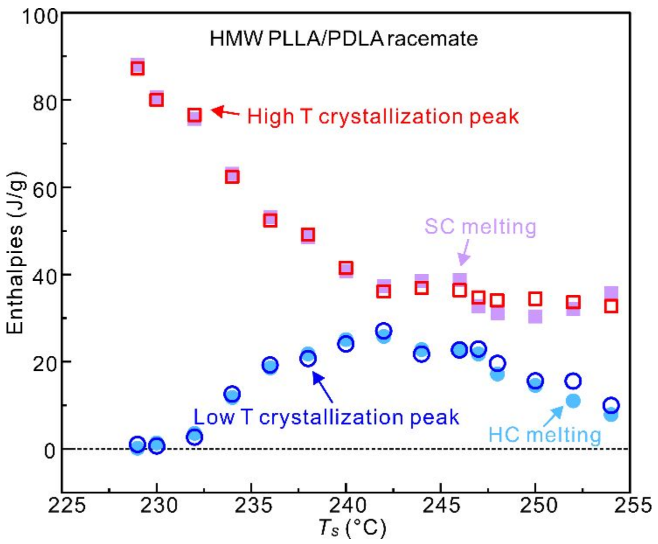

Figure S5. Enthalpies of exotherms on cooling and endotherms on heating versus T_s. The data were obtained from DSC thermograms of HMW PLLA/PDLA racemate.

5. Assignment of exotherm of SC and HC in HMW racemate with cooling rate of 10 and 20 K/min from T_s of 247 ˚C

For HMW PLLA/PDLA racemate, the crystallization peaks of SC and HC (α+α') are partially overlapped when the cooling rate is 10 and 20 ˚C/min. The crystallization enthalpies of SC and HC at these two cooling rates were obtained by peak fitting with modified Gaussian components, as shown in **Figure S6**.

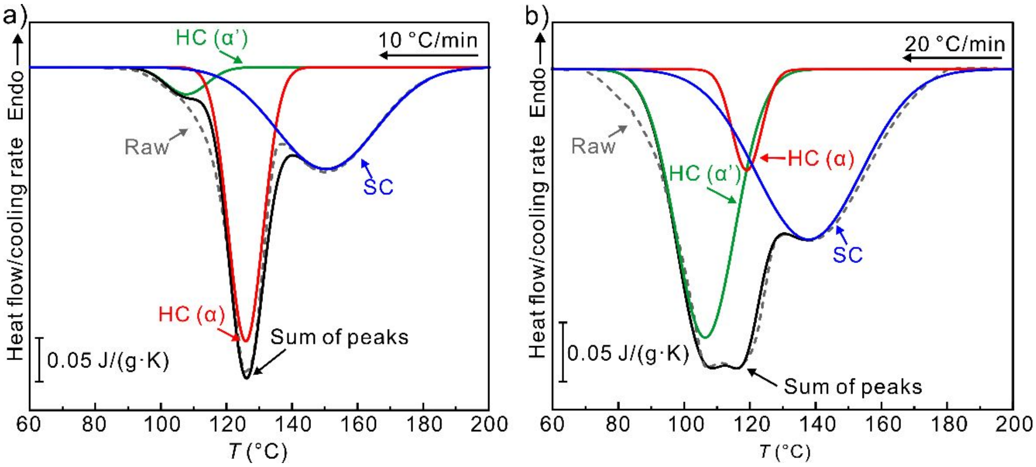

Figure S6. Resolution of HC and SC exotherms by peak fitting for HMW racemate with cooling rate of (a) 10 K/min and (b) 20 K/min.

6. Assignment of SC and HC crystallization in HMW PLLA/PDLA racemate + 1 wt% TMB-5 with $T_s \geq 248$ ˚C

The enthalpies of crystallization and melting of HMW PLLA/PDLA racemate + 1 wt% TMB-5 were measured. Analogous to HMW PLLA/PDLA racemate, the higher and lower crystallization exotherms corresponds to SC and HC (α+α'), respectively. **Figure S7** shows the decomposition of exotherms for $T_s \geq 248$ ˚C. Enthalpies as a function of T_s are shown in **Figure S8**.

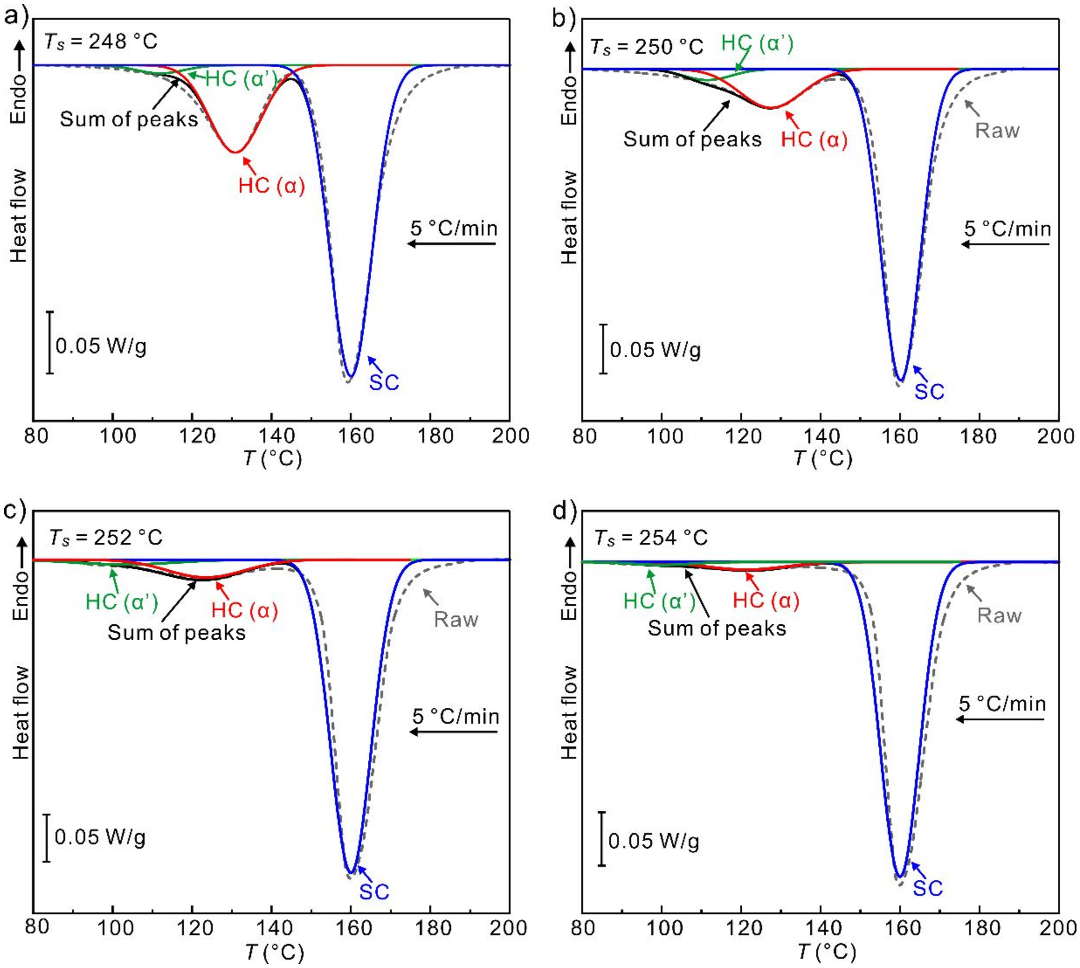

Figure S7. Resolution of exotherm of HMW PLLA/PDLA racemate + 1 wt% TMB-5 with T_s of (a) 248, (b) 250, (c) 252 and (d) 254 ˚C.

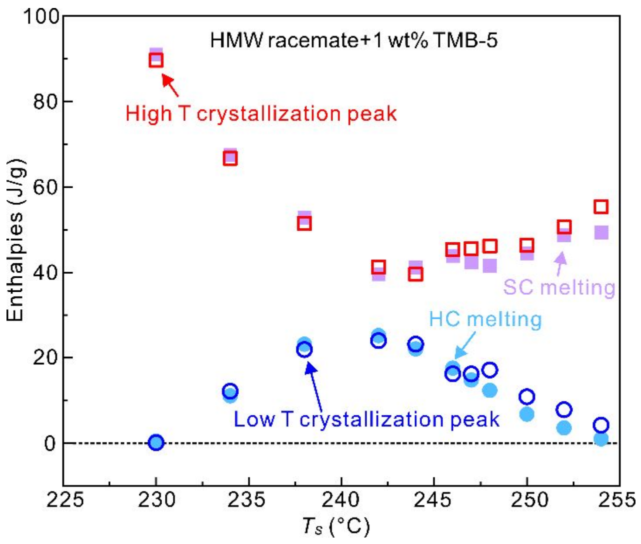

Figure S8. Enthalpies of exotherms on cooling and endotherms on heating for different T_s. The data were from DSC of HMW PLLA/PDLA racemate + 1 wt% TMB-5.

7. Effect of TMB-5 on crystallization of HMW PLLA/PDLA racemate

The crystallization temperature of SC and HC in HMW PLLA/PDLA racemate + 1 wt% TMB-5 were compared with that in neat HMW PLLA/PDLA racemate. **Figure S9** shows that crystallization temperature of SC and HC were both improved with addition of TMB -5 when sample was completely melted ($T_s \geq 248$).

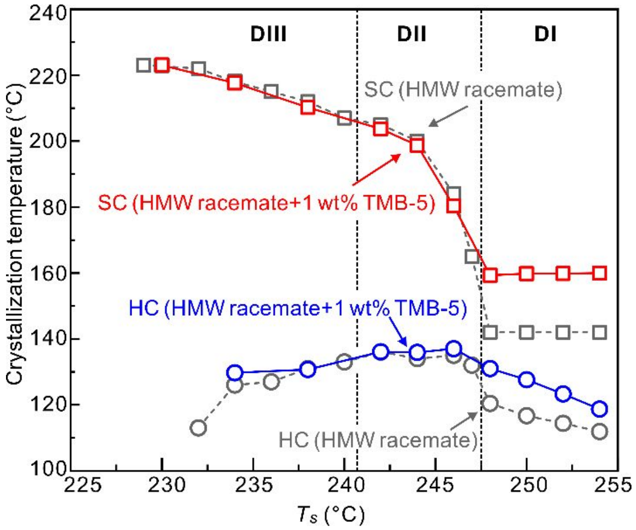

Figure S9. Comparison of crystallization temperature of SC and HC in HMW PLLA/PDLA racemate before and after added 1 wt% TMB-5.

8. POM observation of HMW PLLA/PDLA racemate after annealing at three selected T_s

Crystalline morphologies of HMW PLLA/PDLA racemate annealed at T_s of 236 ˚C, 246 ˚C and 250 ˚C, were studied by POM. No birefringent texture can be observed at the beginning of cooling for the selected three different T_s, as shown in **Figure S10**.

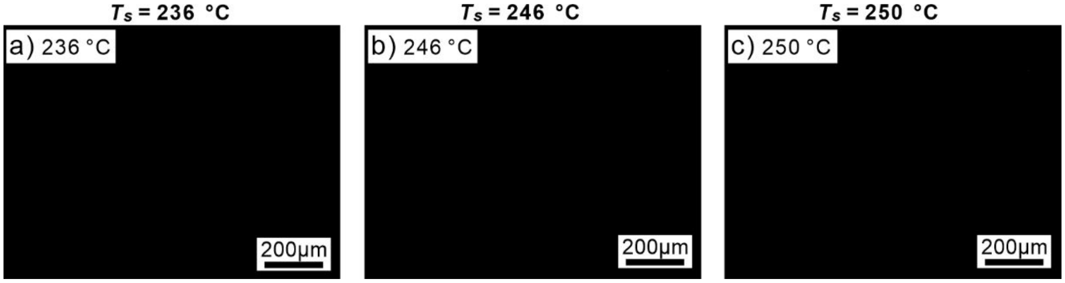

Figure S10. POM micrographs of HMW racemate collected after annealing at different T_s for 2 min.

References

S1 Qiao, Y.; Wang, Q.; Men, Y., Kinetics of nucleation and growth of form II to I polymorphic transition in polybutene-1 as revealed by stepwise annealing. *Macromolecules* **2016,** *49*, 5126-5136.

S2 Alfonso, G. C.; Azzurri, F.; Castellano, M., Analysis of calorimetric curves detected during the polymorphic transformation of isotactic polybutene-1. *J. Therm. Anal. Calorim.* **2001,** *66*, 197-207.
